# Single Nucleotide Recognition and Mutation Site Sequencing Based on a Barcode Assay and Rolling Circle Amplification

**DOI:** 10.3390/bios14110521

**Published:** 2024-10-25

**Authors:** Linmin Zhong, Huiping Chen, Shuang Cao, Shanwen Hu

**Affiliations:** 1 Department of Health Inspection and Quarantine, School of Public Health, Fujian Medical University, Fuzhou 350122, China; 2240210186@fjmu.edu.cn (L.Z.); chp2240210259@fjmu.edu.cn (H.C.); 2 Key Laboratory of Analysis and Detecting Technology, College of Chemistry, Food Safety MOE, Fuzhou University, Fuzhou 350002, China; 231320101@fzu.edu.cn

**Keywords:** single nucleotide polymorphism, barcode, rolling circle amplification, *Helicobacter pylori*

## Abstract

Single nucleotide polymorphisms (SNPs) present significant challenges in microbial detection and treatment, further raising the demands on sequencing technologies. In response to these challenges, we have developed a novel barcode-based approach for highly sensitive single nucleotide recognition. This method leverages a dual-head folded complementary template probe in conjunction with DNA ligase to specifically identify the target base. Upon recognition, the system triggers rolling circle amplification (RCA) followed by the self-assembly of CdSe quantum dots onto polystyrene microspheres, enabling a single-particle fluorescence readout. This approach allows for precise base identification at individual loci, which are then analyzed using a bio-barcode array to screen for base changes across multiple sites. This method was applied to sequence a drug-resistant mutation site in *Helicobacter pylori* (*H. pylori*), demonstrating excellent accuracy and stability. Offering high precision, high sensitivity, and single nucleotide resolution, this approach shows great promise as a next-generation sequencing method.

## 1. Introduction

Single nucleotide polymorphisms (SNPs)—one of the most common forms of genetic variations in the human genome—play an important role in the occurrence and development of human diseases, and their identification is crucial for the study of diseases, pharmacology, and biology [1,2]. Accurate identification of these SNPs is thus essential for advancing our understanding of disease mechanisms, pharmacogenomics, and molecular biology. Traditional SNP detection methods predominantly rely on sequencing technologies, which, while powerful, demand sophisticated equipment and complex data analysis, making them less accessible for routine diagnostics [3]. The programmability and high information density inherent to DNA molecules has spurred the development of innovative DNA-based biosensing technologies, which show significant potential for SNP detection [4,5]. For instance, Xie et al. designed a competitive DNA probe capable of distinguishing single nucleotide variations (SNVs) from wild-type sequences, employing duplex-specific nuclease to achieve specific recognition of mutant sequences [6]. Similarly, Zhou et al. utilized short-chain hybridization combined with capillary electrophoresis to capture and detect SNPs [7], while Xie et al. introduced a bulge-loop into duplex DNA probes to modulate free energy differences, enhancing the specificity of SNV detection [8]. Chen et al. further advanced the field by designing a catalytic hairpin assembly (CHA) biosensor that leverages double base mismatches to achieve highly sensitive detection of mutation targets [9].

A major challenge in SNP detection is achieving both high precision and high sensitivity, especially when detecting low-frequency mutations or analyzing complex biological samples. Many current methods excel in either sensitivity or precision but struggle to balance both, which is crucial for reliable SNP identification [10]. For example, approaches that enhance specificity often do so at the cost of reduced sensitivity, and vice versa, complicating the development of universally applicable detection strategies. Though efforts have been made, the detection of SNPs, particularly in complex biological samples or at low frequencies, remains challenging due to the interference of wild-type sequences. Approaches with high sensitivity and high accuracy are still urgently needed.

Barcoding technology, which simplifies complex information into manageable patterns, has also shown promise in SNP detection [11]. Barcodes can be encoded in various forms—spectroscopic, graphical, electronic, and physical—allowing for flexible applications across different detection platforms [12,13,14,15]. For instance, Zhang et al. combined biological barcodes with catalytic hairpin assembly, using nanoparticles to generate a fluorescence signal for precise miRNA detection [16]. Song et al. further demonstrated the potential of barcodes by integrating metal-organic frameworks (MOFs) with CRISPR/Cas12a systems, achieving ultrasensitive detection of miRNA and protein biomarkers [17,18]. Given the unique sequence-specific recognition capabilities of barcodes, they can be engineered to accurately distinguish between different genetic variants, including SNPs [19,20]. Quantum dots (QDs), semiconductor-based nanomaterials, have emerged as ideal signal labels due to their exceptional signal generation and delivery properties, which are particularly useful in biomedical diagnostics [21,22]. Zhang et al. developed a QDs-mediated fluorescence resonance energy transfer (FRET)-based nanosensor integrated with rolling circle amplification (RCA) for the sensitive detection of SNPs in cancer cells [23]. However, the nanoscale nature of these signals poses challenges for direct observation with conventional microscopy. Moreover, while barcodes offer a powerful means of encoding and biosensing, their application in SNP detection is still in the exploratory stages.

In the present study, we aim to overcome these challenges by developing an innovative barcode sequencing strategy. By using a padlock probe and T4 DNA ligase, we ensure that only the DNA sequences with specific mutations form stable cyclization structures, enabling accurate mutant identification. The rolling circle amplification then significantly increases the target DNA copy number, enhancing detection sensitivity. We employ polystyrene (PS) microspheres as macroscopic signal amplification carriers coupled with the fluorescence recovery of CdSe QDs as the signal output, visualizing the results at the single particle level. Additionally, a barcode screening assay is designed to realize the sequencing at different sites, opening new avenues for research and application in the field of single nucleotide resolution.

## 2. Materials and Methods

### 2.1. Instrumentations and Materials

Phosphate buffered saline (PBS, 0.1 M, pH = 7.4) and sodium borohydride (NaBH_4_) were purchased from Aladdin Co., Ltd. (Shanghai, China). Cadmium chloride (CdCl_2_), selenium powder, and 3-Mercaptopropionic acid (3-MAP) were purchased from Macklin Biochemical Co., Ltd. (Shanghai, China). Polystyrene microspheres (5 µm diameters) were obtained from Xianfeng Nanomaterial Technology Co., Ltd. (Nanjing, China). N-(3-(Dimethylamino) propyl)-N′-ethyl carbo-diimide hydrochloride (EDC) and N-Hydroxysuccinimide (NHS) were purchased from Sinopharm Chemical Reagent Co., Ltd. (Shanghai, China). T4 DNA Ligase, Phi 29 polymerase and Nb.BbvCI endonuclease were purchased from New England Biolabs Co., Ltd. (Beijing, China). All oligonucleotides were synthesized by Sangon Biotechnology Co., Ltd. (Shanghai, China). The sequences are listed in supporting information (Table S1). Other chemicals were of analytical grade and were used without further purification. The instruments used in the experiment are listed in the supporting information (Table S2).

### 2.2. Optimizing Conditions for RCA Loop Formation and Amplification

The 100 nM template-A was mixed with the 100 nM target-A and heated in a thermostatic heater at 95 °C for 10 min. One portion was immediately placed on ice to cool down naturally to about 30 °C, and the other portion was cooled down to 30 °C with a metal bath at a rate of −0.5 °C/min to form a loop structure. T4 DNA ligase was then added to final concentrations of 2, 4, and 6 U/mL and incubated for 1 h at 16 °C. The samples were loaded into a 12% Sodium dodecylsulphate polyacrylamide (SDS-PAGE) gel and separated by electrophoresis at 100 V for 1 h.

The amplified products of RCA were characterized by atomic force microscopy (AFM) and transmission electron microscopy (TEM).

After that, Phi 29 polymerase at a final concentration of 0.5 U/mL was added to the ligation product and incubated for 2 h at 30 °C. An occupying strand at a final concentration of 1 μM was added to the amplification products and incubated at room temperature for 1 h to form double strands. Nb.BbvCI endonuclease at a final concentration of 1 U/mL was added to the double-stranded amplification products formed, which was incubated at 37 °C for 1 h and then heated at 80 °C for 20 min. The samples were loaded into a 12% SDS-PAGE gel and separated by electrophoresis at 100 V for 1 h.

### 2.3. Fabrication of the CdSe/PS Nanocomposite

First, 30 mg of selenium powder and 57 mg of NaBH_4_ were dissolved in 15 mL of ultrapure water at 80 °C to obtain the selenium precursor (NaBH_2_Se_2_) solution. A total of 460 mg of CdCl_2_, 260 μL of 3-MAP, and 30 mL of ultrapure water were added to the double-necked bottle, and the pH was adjusted to 8–8.5 with sodium hydroxide (NaOH) solution. After 20 min of nitrogen in the bottle, the selenium precursor system was added and stirred for 1 h at 120 °C (under nitrogen protection). After centrifugation at 12,000 rpm for 20 min, the precipitation was frozen at −40 °C and 100 Pa for lyophilization to complete the preparation of CdSe QDs and conserve it at −4 °C.

A final concentration of 1 μm Hairpin, 10 mM EDC and 10 mM NHS were added to 1 mg/mL CdSe QDs and the reaction was left at room temperature for 1 h to modify the Hairpin. Then PS microspheres with a final concentration of 100 μg/μL were added to CdSe QDs and stirred at room temperature for 8 h. We then washed the sediment with ultrapure water, dispersed and separated it with centrifugation, and conserved the final precipitation product in 1 × PBS, completing the preparation of the CdSe/PS nanocomposite.

The morphological structure and spectral properties of the CdSe/PS nanocomposite were characterized by high resolution transmission electron microscopy (HRTEM), X-ray polycrystalline diffractometry (XRD), Fourier transform infrared spectrometry (FTIR), ultraviolet-visible spectrometry (UV-VIS), and fluorescence spectrometry (FLS).

### 2.4. Validation of the CdSe/PS Nanocomposite for Single Base Resolution Performance

A series of 10 μL saliva samples with different target concentrations (0.1, 1, 10, and 100 pM) were incubated with 90 μL of the CdSe/PS nanocomposite solution. Fluorescence scanning was performed using a laser confocal scanning microscope (EX = 405 nm, EM = 561 nm). The fluorescence intensity was analyzed by NIS-Viewer 4.2.0 software to verify the target concentration-dependent fluorescence recovery of the CdSe/PS nanocomposite.

The 10 μL saliva samples at different target concentrations (0.1, 1, 10, and 100 pM) and 10 μL of two single base unpaired samples were incubated by adding 90 μL of CdSe/PS nanocomposite solution to validate its detection of target single-base mutations.

Target strands in different concentrations were added to 200 μL saliva samples, and the concentration was determined by this method, then recoveries were calculated to investigate the accuracy and validity of this method in a real environment.

*Helicobacter pylori* (*H. pylori*)-positive saliva samples were analyzed following this approach, which was compared with the standard PCR method to verify the true validity (Table S3). *H. pylori*-positive saliva samples were obtained from volunteers in the First Affiliated Hospital of Fujian Medical University, and this research was approved by the Institutional Ethics Committees of Fujian Medical University (Certificate Number: 2022-15).

### 2.5. Statistics Section

In this study, all spectral intensities, unless otherwise specified, are the average values of three repeated measurements. The fluorescence imaging results are all original fluorescence photographs. For the particle intensities in the fluorescence imaging results, we use the Image J 2.14.0 software to select 10 representative particles in the image, perform chromaticity analysis to obtain the pixel intensities, and take the average to be recorded as the fluorescence imaging detection intensity of the image. *p*-values are obtained by Wilcoxon test.

## 3. Results and Discussion

### 3.1. The Principle of the Barcode-Based Single Base Mutation Sequencing

**Figure 1:**
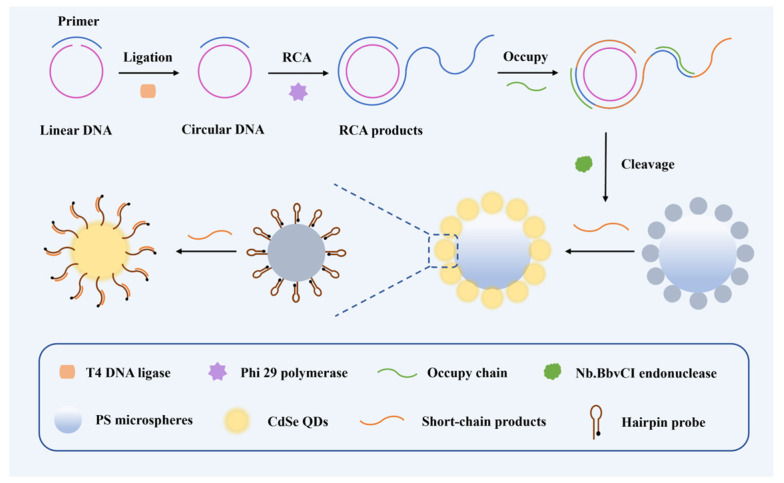
Illustration of single base mutation detection and RCA amplification target sequence and preparation of the CdSe/PS nanocomposite.

The biosensing mechanism consists of three stages, as illustrated in Figure 1. The first stage is the identification of mutated bases. A bifunctional probe was designed to target a specific nucleic acid sequence. This probe can hybridize with both ends of the target strand, bending and converging at the mutation site [24]. If a mismatch occurs at this site, the probe is unable to form a closed loop, preventing further reactions. Only when the target strand is fully complementary to the probe will a complete double strand form, allowing DNA ligase to ligate the strands, thus closing the probe and triggering stage 2, a rolling circle amplification (RCA) reaction. This RCA process produces long-chain products with repetitive sequences [25,26]. These products are then cleaved by the endonuclease Nb.BbvCI, generating multiple copies of the target sequence and completing signal amplification [27]. Stage 3 is a nanomaterials based fluorescent readout, the resulting short-chain products interact with a hairpin probe-modified CdSe nanoparticle, causing the hairpin structure to unfold and separating the quenching group from the CdSe, thereby restoring the fluorescence signal. CdSe nanoparticles self-assemble on the surface of polystyrene (PS) beads through electrostatic adsorption, making the fluorescence signal detectable under a microscope. This process allows the creation of a barcode matrix for fluorescent screening of mutation sites.

### 3.2. Single Nucleotide Recognition and RCA Amplification of Target Nucleic Acids

The key to single nucleotide recognition in nucleic acid reactions is to establish a unique thermodynamic position for the mutation site. This can be achieved by placing the mutation site at a critical location, such as the toehold region that initiates the nucleic acid reaction, as used in short-chain effect-based biosensing. In this design, we constructed a bifunctional, bent structure with the mutation site located at the junction of the bend. The action of DNA ligase makes the matching at the mutation site essential for the bifunctional probe to close the loop, thereby facilitating the recognition of single nucleotide mutations.

**Figure 2:**
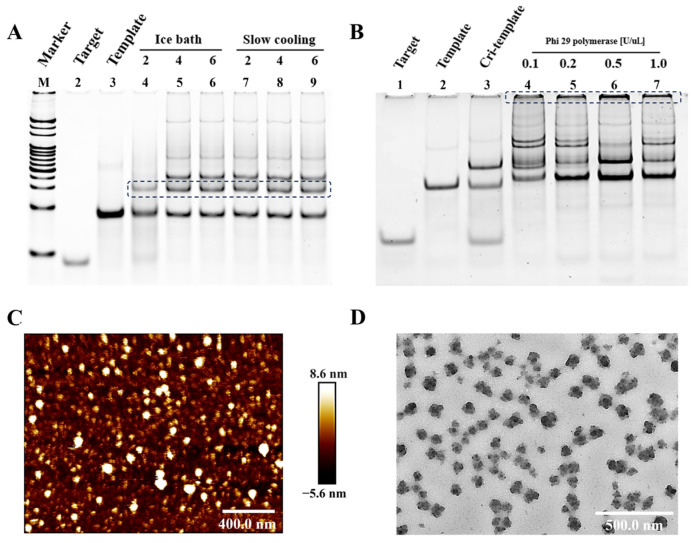
Optimization and validation of the RCA reaction. Gel electropherogram image of condition optimization for RCA loop formation (**A**) and amplification (**B**). All samples were loaded at 10 μL, marker was 20 bp, buffer solution was 10 mM Tris-Borate-EDTA buffer (TBE, pH 8.2–8.4), and voltage and time were 100 V and 70 min, respectively. (**C**) ATM characterization and (**D**) TEM characterization of RCA products of target-A.

We begin by optimizing the conditions for DNA ligase. For complete matching at the mutation site, two annealing methods were tested: rapid cooling using an ice bath and slow cooling, each with three different enzyme concentrations. As shown in Figure 2A, lanes 2 and 3 display template-A and target-A, respectively, where single bands are visible at the corresponding positions in each lane. Lanes 4, 5, and 6 represent varying T4 DNA ligase concentrations under ice bath conditions, while lanes 7, 8, and 9 represent different concentrations under slow cooling. Notably, lane 4 has the least nonspecific bands, indicating the highest circularization efficiency. Thus, the conditions used in lane 4 (ice bath cooling and 2 U/μL T4 DNA ligase) were chosen as optimal for single nucleotide recognition.

Next, we optimized the conditions for the RCA reaction, focusing primarily on the activity of Phi 29 polymerase. As shown in Figure 2B, lanes 1 and 2 represent template-A and target-A, respectively, where single bands appear at the expected positions. Lane 3 depicts the loop formed under the optimal conditions identified from the previous step. Lanes 4 through 7 display the results of different Phi 29 polymerase concentrations (0.1, 0.2, 0.5, and 1 U/μL). Analysis of these lanes reveals that while all show amplified bands at the top, lane 6 (0.5 U/μL Phi 29 polymerase) exhibits the best performance, indicating the highest amplification efficiency. As a result, a concentration of 0.5 U/μL Phi 29 polymerase was determined to be optimal for the RCA reaction.

To further characterize the RCA products, we used atomic force microscopy (AFM) and transmission electron microscopy (TEM). After desalination pretreatment, AFM images revealed bright white particles that were uniformly dispersed, with particle sizes ranging from approximately 30 to 40 nm and a height of 6 to 8 nm. Subsequently, we performed negative staining of the RCA products with phosphotungstic acid for TEM observation. TEM images showed distinct black spherical particles, approximately 35 nm in diameter, evenly distributed without any signs of agglomeration. These morphological observations are consistent with those reported in the literature, further confirming the successful formation of RCA products [28].

After confirming the successful amplification of RCA, we used the enzyme Nb.BbvCI to digest the long-chain products into multiple short single strands, allowing for easier binding of the products to the subsequent quantum dot (QD) composites. As shown in Figure S1, lanes 2 and 3 represent template-A and target-A, respectively, where distinct single bands are visible in each lane. In lane 5, the hybridization of template-A and target-A forms a circular structure, indicated by a band of lower mobility, confirming the presence of a stable circular product. Lane 6 displays the amplified product, while lane 7 shows the product after the addition of the Nb.BbvCI enzyme post-amplification. Following digestion with Nb.BbvCI, bands corresponding to the expected size of the digested strands are visible at the bottom of lane 7, confirming the successful generation of RCA digestion products. These digested products will be further employed to activate the fluorescence signal in the nanocomposite materials.

### 3.3. Construction of the CdSe/PS Nanocomposite and Fluorescence Signal Readout System

**Figure 3:**
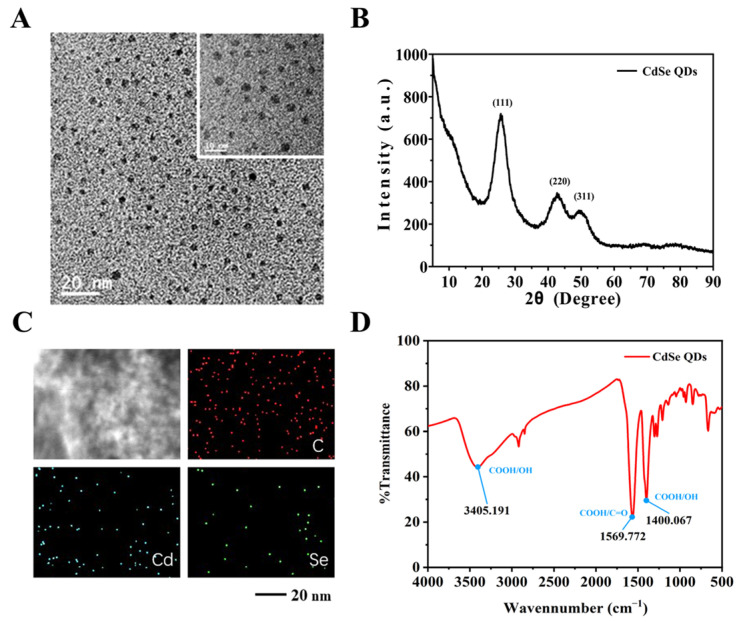
Morphology and structure analysis of CdSe QDs. (**A**) High-resolution TEM diagram, (**B**) X-ray diffraction (XRD) patterns, (**C**) elemental distribution diagram, and (**D**) infrared spectrum diagram of CdSe QDs.

We employed CdSe as the signal source for the detection system due to its excellent fluorescence properties and dispersion [29]. HRTEM characterization, shown in Figure 3A, reveals that the synthesized material has a relatively uniform spherical shape with a size of approximately 3–5 nm, meeting the size requirements for QDs. The particles are also uniformly dispersed, with no significant agglomeration observed. To investigate the phase properties of the synthesized material, we measured the XRD patterns (Figure 3B). Strong diffraction peaks appear at diffraction angles of 25°, 43°, and 50°. By comparing these with standard patterns, these peaks correspond to the (111), (220), and (311) crystal planes of MPA-CdSe quantum dots, indicating that the prepared nanoparticles have a cubic crystalline structure. Furthermore, to explore the elemental distribution of CdSe QDs, we performed elemental mapping using scanning electron microscopy (SEM) (Figure 3C). The results show a uniform distribution of Cd and Se elements with a high degree of overlap, confirming the successful synthesis of CdSe QDs. Finally, since surface carboxyl groups are essential for subsequent functional modifications, we investigated the surface functional group distribution using infrared spectroscopy. From Figure 3D, an asymmetric stretching peak of the C=O bond in the -COOH group is observed at 1569 cm^−1^, and a peak corresponding to the -OH group of the COOH group appears at 1400 cm^−1^, strongly confirming the presence of surface carboxyl groups on the CdSe QDs.

**Figure 4:**
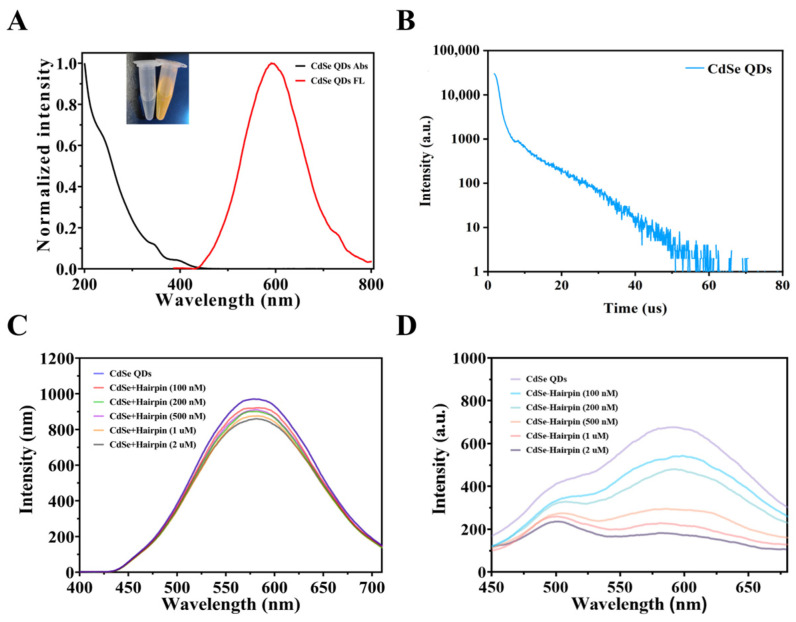
Fluorescence characteristics of CdSe QDs. (**A**) UV absorption/fluorescence spectra diagram and (**B**) fluorescence lifetime diagram of CdSe QDs. Fluorescence spectra of CdSe QDs with (**C**) and (**D**) without EDC/NHS modified hairpin chains at different concentrations.

To investigate the optical properties of CdSe QDs, we first measured the UV spectrum. As shown in Figure 4A, the UV absorption spectrum spans 200–400 nm, indicating that the synthesized material can be excited by almost the entire UV spectrum. We selected 365 nm as the excitation wavelength for the material, and its emission wavelength is shown by the red line in Figure 4A. The fluorescence emission range appears between 400–800 nm, with a maximum emission peak around 600 nm and a Stokes shift of nearly 200 nm, demonstrating excellent fluorescence properties. Subsequently, to further verify the persistence of the fluorescence emission, we measured the fluorescence lifetime curve of CdSe QDs using an FLS 980 fluorometer. As shown in Figure 4B, the decay curve is on the nanosecond (ns) scale. After fitting the decay curve, the fluorescence lifetime was calculated using the second-order fitting curve’s fluorescence lifetime formula to be 143 ± 39 ns. The characterization of CdSe QDs above demonstrates that it is an excellent fluorescent nanomaterial, beneficial for the construction of subsequent composite materials and the biosensing process.

We constructed a fluorescence signal readout system based on CdSe. First, we covalently linked hairpin probes modified with quenching groups to CdSe to form a FRET system, establishing an initial signal in the quenched state. Using EDC/NHS catalysis, we linked the -COOH of QDs with the -NH_2_ of the hairpin chain. As shown in Figure 4C, in the absence of the EDC/NHS catalyst, the fluorescence intensity of CdSe QDs does not quench with increasing hairpin chain concentration (100, 200, 500, 1000, and 2000 nM). This is because CdSe QDs and the hairpin are in a free state and are too far apart for effective FRET to occur. However, under EDC/NHS catalysis, as shown in Figure 4D, the fluorescence intensity of CdSe QDs gradually quenches with increasing hairpin concentration (100, 200, 500, 1000, and 2000 nM), indicating the successful modification of the hairpin chain on CdSe QDs and the formation of an effective FRET system. This system can be used for a subsequent signal readout of the product chain.

**Figure 5:**
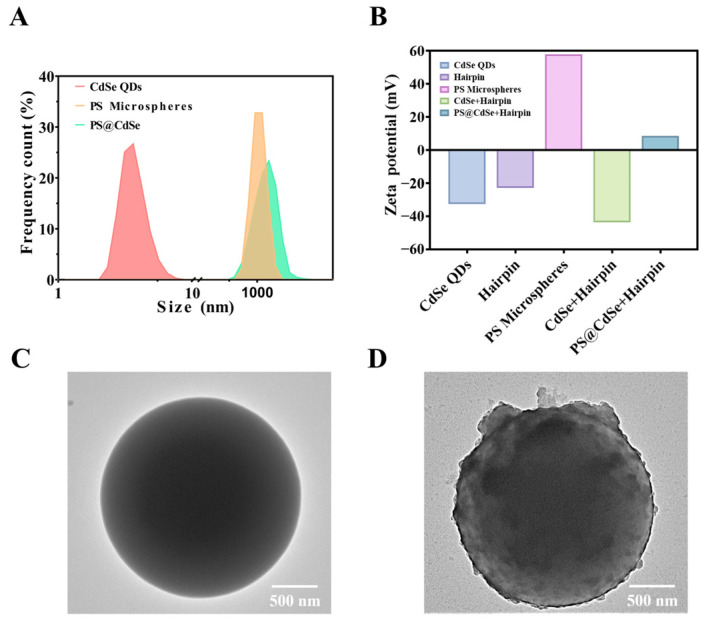
Characterization of the CdSe/PS nanocomposite. (**A**) Particle size diagram and (**B**) Zeta-electric potential diagram of CdSe/PS nanocomposite assembly. Transmission electron microscopy diagram of (**C**) PS microspheres and (**D**) the CdSe/PS nanocomposite.

The particle size of CdSe QDs is at the nanometer scale, making it impossible to directly observe the particles under a fluorescence microscope. By self-assembling onto PS microspheres through electrostatic adsorption, the particle size can be increased, forming a particle visible under the microscope for fluorescence detection. We first measured the particle sizes of CdSe QDs, PS microspheres, and PS@CdSe QDs separately. As shown in Figure 5A, the particle size distribution shifts to the right when PS microspheres are modified with CdSe QDs, indicating the formation of larger composite materials. The potential changes during the assembly process are shown in Figure 5B. The negatively charged CdSe QDs remain negatively charged after binding with the negatively charged hairpin chain, while the PS microsphere surface is positively charged. After electrostatic adsorption and binding, the composite material presents a positive charge. The corresponding potential changes also indicate the successful layer-by-layer assembly of the CdSe/PS nanocomposite. Finally, to observe the material more intuitively, we used TEM to capture images of PS microspheres and the CdSe/PS nanocomposite. It can be seen that the initial PS microspheres are very regular spherical particles with a particle size of 1 μm. After adsorbing CdSe QDs with hairpin modification, the PS microspheres have obvious particle encapsulation, and the particle size is significantly increased, thus more intuitively verifying the formation of the CdSe/PS nanocomposite QDs.

### 3.4. CdSe/PS Nanocomposite Detection Performance Evaluation and Applications

**Figure 6:**
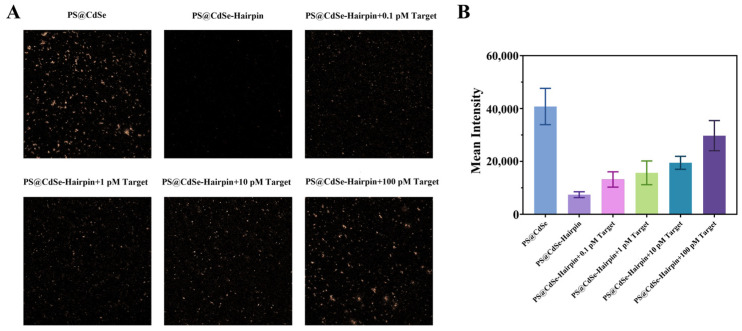
Detection performance of the target sequence by the CdSe/PS nanocomposite. (**A**) Confocal images of the CdSe/PS nanocomposite for detection of target-A with different concentrations. (**B**) Quantitative analysis of confocal fluorescence intensity (*n* = 3).

We applied the constructed nucleic acid reaction system and composite nanomaterials to the detection of mutation sites in *H. pylori*. First, a quantitative model was established using a perfectly matched probe and target. The concentration of target-A was set to 0.1, 1, 10, and 100 pM. After RCA reaction and enzymatic digestion of the target strand A, the CdSe/PS nanocomposite exhibited particle-like fluorescence under a confocal microscope, as shown in Figure 6A. Additionally, intensity analysis using software revealed that the fluorescence signal increased with the increase of target concentration, as shown in Figure 6B.

**Figure 7:**
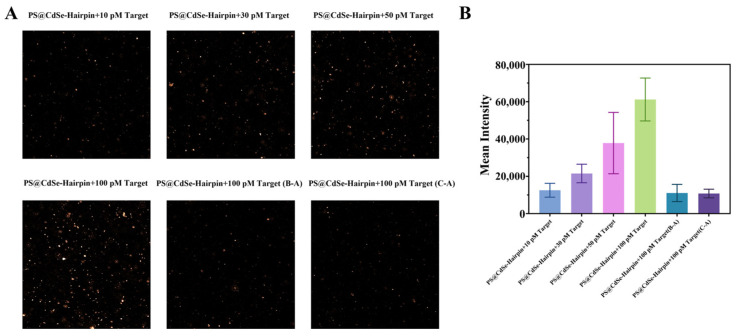
Identification performance of single base mutation by the CdSe/PS nanocomposite. (**A**) Confocal images of the CdSe/PS nanocomposite for detection of non-mutated target and single-base mutated. (**B**) Quantitative analysis of confocal fluorescence intensity (*n* = 3).

Next, we explored the method’s ability to recognize single-base mutations. The wild-type of *H*. *pylori* was used as target A, and the A2143G and A2142G mutations of Clarithromycin were set as target B and target C, respectively (sequence information is shown in Table S1). We combined non-mutated target, target-A, and single-base mutated target-B and target-C, with the same template, template-A, and performed amplification and confocal imaging. As shown in Figure 7A, the fluorescence of the CdSe/PS nanocomposite was significantly restored only in the group with complete base pairing (Target-A and Template-A). In contrast, the fluorescence in the groups with single-base mismatches did not recover significantly. Fluorescence quantitative analysis of the confocal images, shown in Figure 7B, indicates that the fluorescence intensity of the fully paired group is about ten times that of the single-base mismatch groups. This demonstrates the excellent single-base discrimination capability of the RCA reaction for *H. pylori* single base mutations.

The previous validations were carried out in a simulated solution environment; in order to more realistically verify the effective feasibility of real environment detection, we took some real negative saliva samples [30]. Then different concentrations of target-A were added to this sample solution, after which it was detected by the experimental method described above. The results are shown in Table 1, which show that the average recovery of *H. pylori* DNA in real samples was in the range of 94–105%, which was very close to 100%; and the Relative Standard Deviation (RSD) was also lower than 3.2%, meeting the standard that usually requires between 1% and 5%. The above results indicate that the CdSe/PS nanocomposite has a strong anti-interference ability in detecting single-base mutated target sequences, and can be used for the detection of complex environmental samples, which will have great potential for practical application in early diagnosis.

To evaluate the difference between our method and the traditional standard method (polymerase chain reaction, PCR), we performed both assays on the same positive samples. By adjusting for different concentrations of target sequences, we constructed PCR curves that were linear with concentration (Figure S2A,B). Substituting the cycling threshold (Ct) for PCR into this linear curve, we calculated the concentration of *H. pylori* in saliva samples to be 98 pM. The concentration measured using our method was 95 pM, and the margin of error was tightly controlled to less than 5% compared to the PCR method. This indicates that our method is in high agreement with the internationally recognized standard method (PCR) in terms of the concentration detected, with error values controlled within acceptable limits.

**Figure 8:**
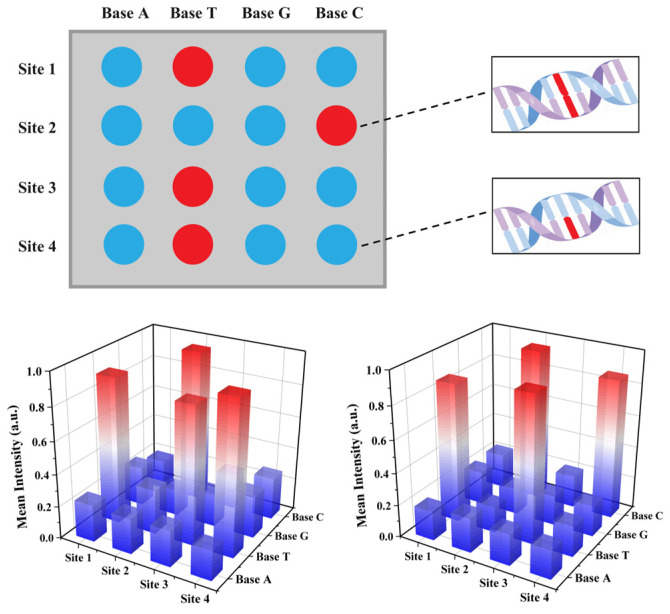
Array-based multiplex detection of single nucleotide resolution.

Building upon the precise single-base identification at the individual site, we have innovatively designed a bio-barcode sequencing array. This approach enables array screening of each sample, allowing base identification at each locus and subsequently aggregating the recognition results to obtain comprehensive sequencing data. As illustrated in Figure 8, during the sequencing process, a template strand containing the bases A, T, C, and G is constructed for each site to assess the sample. The results indicate that the T base at the first locus is a perfect match with the sample, confirming T as the base at this locus. The intersection point is then adjusted to the next base, and the subsequent loci are sequentially detected, allowing for an accurate reading of the complete base sequence of the sample as T, C, T, T. Compared to traditional methods, this barcode sequencing technology offers higher accuracy and sensitivity, with the added advantage of enhanced stability due to fluorescence single-particle imaging. This approach has the potential to be a high-precision sequencing method.

## 4. Conclusions

In summary, we innovatively combined the assay method with the barcode; the specific padlock probe can recognize SNPs while amplifying the signal through RCA and expanding the scale of the result visualization carrier. The strategy shows good performance in accuracy, sensitivity, and detection selectivity. The approach achieved the detection of SNPs and also accomplished the analysis of artificial samples that mimic complex situations. A single nucleotide resolution sequencing for *H. pylori* DNA mutants is realized on a barcode array, showing great potential for future sequencing techniques.

## Figures and Tables

**Table 1:** Recovery of standard samples added to saliva sample.

Sample	Initial Concentration (pM)	Fortified Concentration (pM)	Total Concentration (pM)	Recovery (%)	RSD (*n* = 3, %)
Saliva	0.0	10.0	10.3	104.1%	2.43%
Saliva	0.0	25.0	25.5	101.7%	1.55%
Saliva	0.0	50.0	48.7	97.7%	3.20%

## Data Availability

Dataset available on request from the authors.

## Supplementary Materials

The following supporting information can be downloaded at: https://www.mdpi.com/article/10.3390/bios14110521/s1, Table S1. DNA sequences used in this experiment; Table S2. Main apparatus used in this experiment; Table S3. PCR amplification system; Figure S1. Gel electrophoretic verification of digestion of RCA amplification products; Figure S2. Validation of assays by standard PCR methods.
