# Supplementary material for: Single Nucleotide Recognition and Mutation Site Sequencing Based on a Barcode Assay and Rolling Circle Amplification
Source: Biosensors (Basel). 2024 Oct 25;14(11):521. doi: 10.3390/bios14110521 (PMC11592053; doi:10.3390/bios14110521)
Supplement: Supplementary file 1 [file biosensors-14-00521-s001.zip › biosensors-3257691-supplementary.pdf]

Single Nucleotide Recognition and Mutation Site Sequencing based on a Barcode Assay and Rolling Circle Amplification

Linmin Zhong [1], Huiping Chen [1], Shuang Cao [2], Shanwen Hu [1,*]

Table S1. DNA sequences used in this experiment
Table S2. Main apparatus used in this experiment
Table S3. PCR amplification system
Figure S1. Gel electrophoretic verification of digestion of RCA amplification products
Figure S2. Validation of assays by standard PCR methods

Table S1. DNA sequences used in this experiment

DNA	Sequence (5'-3')
Target A	TCCACGGGGTCTTTCCGTCTTGCCGC
Template A	AAGACCCCGTGGAACAGAGCAAGTCCGTCTTGCCCTC AGCATGCATGCGGCAAGACGGA
Target B	TCCACGGGGTCTCTCCGTCTTGCCGC
Target C	TCCACGGGGTCTTCCCGTCTTGCCGC
Occupy	GCCCTCAGCATGCAT
Hairpin	NH2-GGAACAGAGCAAGTCCGTCTTAAGCTCTGTTCC-BHQ2
Forward primer	TTCAACGCTTCATCTTGCGACTGACGCATT
Reverse primer	ATACCACCCTTGATGTTTCTGTTAGCTAAC

Table S2. Main apparatus used in this experiment

Apparatus	Specification/Model	Manufacturer
Pure water/ultrapure water integrated system	Milli-Q Integarl	Millipore
Constant temperature metal bath	ThermoStat plus	Eppendorf
Transmission electron microscopy	HT7700	Hitachi
Gel Imaging InstrμMent	ChemiDoc ™ Touch	Bio-Rad
Refrigerated high-speed centrifuge	H1850R	CENCE
Fluorescence spectrometer	Cary Eclipse	Agilent Technologies
Nano Particle Size Potentiometer	Zetasizer Nano ZS	Malvern Panalytical
Ultraviolet-visible spectrometer	UH4150	Hitachi
Multi-function enzyme labeler	Infinite M200 PRO	TECAN
Confocal laser scanning microscopy	FluoviewSIM-A1	Nikon
Constant temperature heating magnetic stirrer	DF-101D	YUHUA

Real-time fluorescence quantitative PCR instrμMent	Millicell ERS-2	Merck Millipore
Dark box ultraviolet analyzer	ZF-20D	YUHUA
Freeze dryer	FD-1A-50	BIOCOOL

Table S3. PCR reaction system

Composition	Volume (μL)
2× superReal PreMix Plus	10
Forward primer (10 μM)	0.6
Reverse primer (10 μM)	0.6
cDNA	5
50×ROX Reference Dye	2
RNase-free ddH2O	1.8

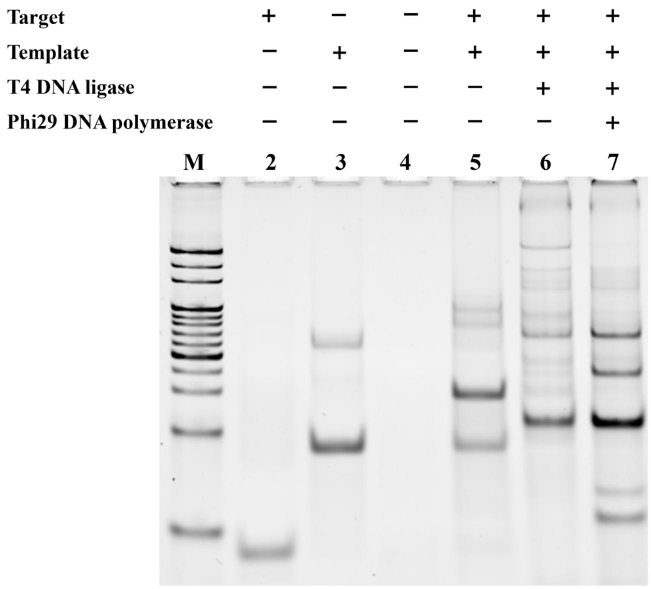

Figure S1. Gel electrophoretic verification of digestion of RCA amplification products

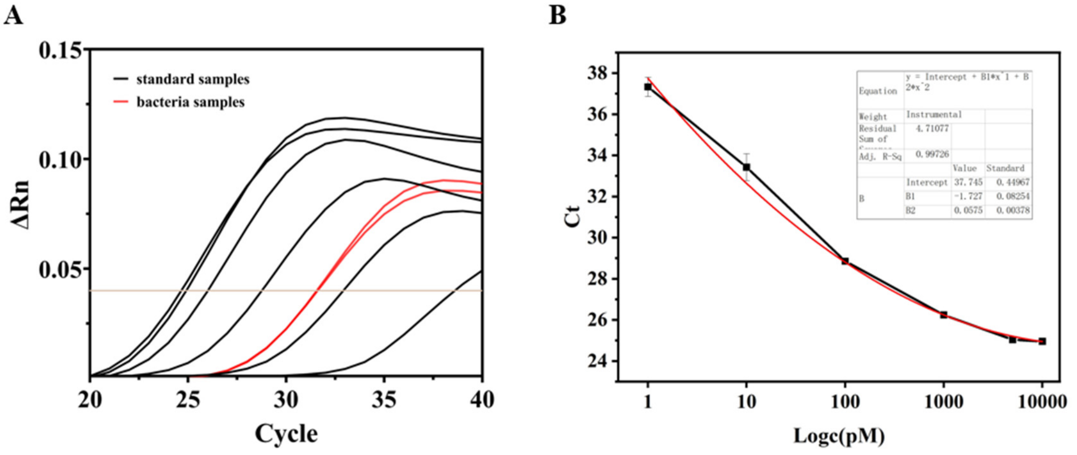

Figure S2. Validation of assays by standard PCR methods. (A) PCR amplification curves of standard samples with different concentrations. (B) Linear curves of standard samples with different concentrations and amplification times. Where the real sample to be measured is in the range of PCR amplification curve, the theoretical concentration value can be obtained by substituting linear curve. (*n*=3)
